# Stepwise evolution of two giant composite LTR-retrotransposon-like elements DA and Xiao

**DOI:** 10.1186/1471-2148-9-128

**Published:** 2009-06-05

**Authors:** Xuanyang Li, Jennifer Slife, Neil Patel, Shaying Zhao

**Affiliations:** 1 Department of Biochemistry and Molecular Biology, Institute of Bioinformatics, University of Georgia, Athens, GA, USA

## Abstract

**Background:**

We recently discovered two composite long terminal repeat (LTR)-retrotransposon-like elements which we named DA (~300 kb) and Xiao (~30 kb), meaning big and small in Chinese respectively. Xiao and DA (three types of DA identified) were found to have been derived from several donor sites and have spread to 30 loci in the human genome, totaling to 5 Mb. Our bioinformatics analyses with the released human, chimp, rhesus macaque, orangutan, and marmoset genomic sequences indicate that DA and Xiao emerged ~25 million years (Myr) ago.

**Results:**

To better understand the evolution of these two complex elements, we investigated various internal junctions of DA and Xiao as well as orthologous genomic sites of the 30 DA/Xiao loci in non-human primates including great apes, lesser apes, Old World monkeys, New World monkeys, and a prosimian. We found that Xiao and type I DA first emerged in the genome between 25 and 18 Myr ago, whereas type II and Type III DAs emerged between 14 and 7 Myr ago. Xiao and DA were most active in great apes, with their amplification peaking during 25-14 and 14-7 Myr ago, respectively. Neither DA nor Xiao seem to have been active in the human and chimp genomes during last 6 Myr.

**Conclusion:**

The study has led to a more accurate age determination of the DA and Xiao elements than our previous bioinformatics analyses, and indicates that the amplification activity of the elements coincided with that of group I HERV-Es during evolution. It has also illustrated an evolutionary path with stepwise structural changes for the elements during past 25 Myr, and in doing so has shed more light on these two intriguing and complex elements that have reshaped our genome.

## Background

Transposable elements (TEs) [1-6] are grouped into retrotransposons and DNA transposons, depending upon whether or not RNA intermediates are required for transposition. Simple DNA transposons such as insertion sequences (IS) encode a transposase and have an inverted repeat of usually 9–41 bp at each end [1,2]. Composite DNA transposons found in bacteria such as Tn10, Tn5, and Tn9 contain a middle region (often encoding a drug resistance gene) flanked by two IS elements. Owing to these flanking IS elements, the middle region of a composite DNA transposon becomes mobile as well. Retrotransposons can be divided further into two groups: one with a long terminal repeat (LTR) of a few hundred to over 1 kb long at each end (e.g., retroviruses) and the other without LTRs (e.g., long interspersed nuclear elements or LINEs) [2]. Retroviruses and LINEs encode reverse transcriptases that are essential for their amplification in the genome. In most cases, insertion of either a DNA transposon or a retrotransposon results in target site duplication (TSD) of usually 2–20 bp [1,2]. While composite DNA transposons such as those in the Tn family have been known for quite some time, a composite retrotransposon with a similar architecture has yet to be discovered.

The human genome contains significantly more recognizable retrotransposons (nearly 50%) than DNA transposons (only 3%) [6-8]. The already identified retrotransposons include LTR elements such as human endogenous retroviruses (HERVs), as well as non-LTR elements such as LINEs (e.g., L1s) and short interspersed nuclear elements (SINEs) (e.g., Alus). Although hundreds of thousands of copies of individual TEs have been identified [7,8], only a small number of composite elements are reported for the human genome including SVA that contains SINE, VNTR (variable numbers of tandem repeats), and Alu elements [9]. Additional examples are the composite DNA transposon Ricksha, as well as Harlequin and HERV39 that have mostly simple repeats inserted into the relevant HERV elements.

Segmental duplications (SDs), another type of repeating DNA which differs from the traditional TEs described above, make up ~5% of the human genome [10]. SDs are low copy number repeats, with a size of at least 1 kb and ≥ 90% sequence identity among the copies. Although under extensive investigation in recent years, the origins and the duplication mechanisms of SDs remain largely unclear [11,12]. No possible linkages between SDs and retrotransposons had been reported until our recent discovery of two composite LTR-retrotransposon-like elements in the human genome [13].

While characterizing a genome-wide SD in the human genome, we discovered two new composite LTR retrotransposon-like elements, which we named DA and Xiao, meaning big and small in Chinese respectively. Complete DA/Xiao copies were found to have an architecture resembling bacterial composite DNA transposon Tn9, consisting of a middle region (the core) flanked by two elements that are direct repeats of each other. The only exception to this is that the flanking element is a modified HERV-E rather than a DNA transposon [13]. However, barring this structural resemblance, there is no experimental evidence that could determine whether DA and Xiao are indeed composite LTR-retrotransposons. Consequently, it is currently unclear whether they have propagated in the genome via retrotransposition or by other mechanisms.

Xiao, with a core of 10 kb, was found to originate from a region of chromosome 19p encoding olfactory receptor 7E members [13-15]. DA, with a core of ~260 kb, appears to have evolved from a Xiao that had an extra LTR5B element, which likely evolved from an ancestral Xiao via insertion of a HERV-K that later degenerated to a solo LTR [13]. The evolvement of DA, according to our analyses [13], was accomplished by insertion of ~200 kb of chimeric sequence derived from chromosomes 16p and 21q into the Xiao core, followed by insertion of 56 kb of TEs and retrogenes. DA or Xiao elements were identified at 30 loci on 12 chromosomes, harboring a total of 20 copies of DA and 45 copies of Xiao [13]. Based on our reconstructed human-mouse-rat ancestral genome [13] and the rhesus macaque genome [16], only DAs mediated intra-chromosomal rearrangements.

**Figure 1:**
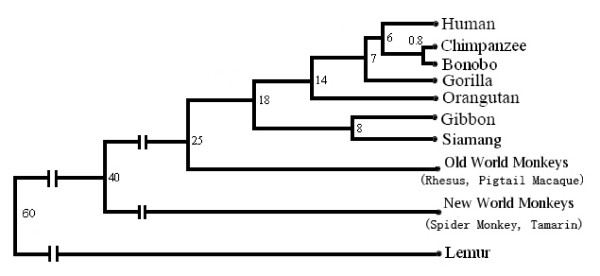
**Primates that were being investigated included great apes (the human, the chimp, the bonobo, the gorilla, and the orangutan), lesser apes (the gibbon and the siamang), Old World monkeys (the rhesus macaque and the pigtail macaque), New World monkeys (the spider monkey and the tamarin), and a prosimian (the lemur)**. The number near each node represents the divergence time in Myr. The scientific name of each species was listed in the legend of Fig. 2.

Our previous bioinformatics analyses [13], with the published human, chimp and rhesus macaque genomic sequences [7,8,16,17] as well as the recently released orangutan and marmoset genomic contig sequences, indicated that DA and Xiao emerged in the genome after the human/apes diverged from Old World monkeys ~25 million years (Myr) ago [18]. In addition, structural analyses indicated three types of DA existing in the genome: type I DA is the most ancestral form (found in the human, chimp, and orangutan genomes); type II has an HERV-E inserted into an existing HERV-H, while type III has a sequence duplication of ~100 kb derived from chromosome 8p and is the most recently evolved (found in the chimp and human genomes only) [13]. To further refine this analysis, we conducted experiments to investigate the Xiao/DA orthologues in primates whose genomes have not yet been sequenced. This study greatly expanded the primate list under examination by including great apes, lesser apes, Old World Monkeys, New World Monkeys, and a prosimian (Fig. 1), covering an evolutionary span of 60 Myr. The analyses led to a more accurate age determination of DA and Xiao, indicating that their amplification activity coincided with that of group I HERV-E elements. It also illustrated an evolutionary path with stepwise structural changes for the elements. The study has shed more light on theses two intriguing and complex elements that have reshaped our genome.

## Results

### Age determination of Xiao as well as type I, type II and type III DAs by examining their internal junctions

#### Duplicon type-specific internal primers

**Figure 2:**
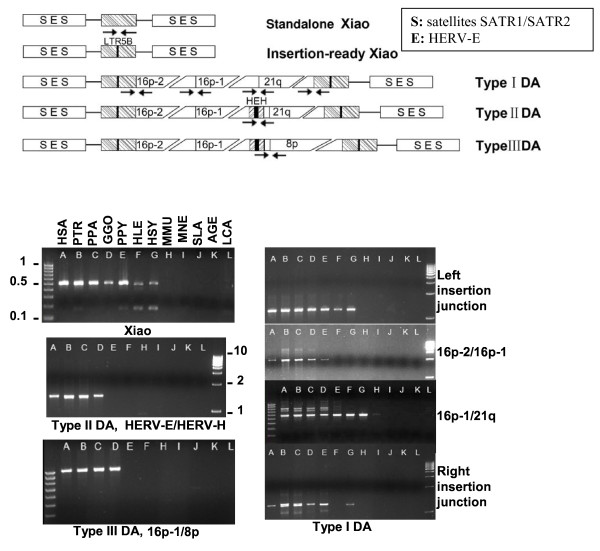
**Age determination of Xiao, type I, type II, and type III DAs**. **Top**: The primer pairs, indicated as paired arrows, flanked the junctions unique to each type of Xiao or DA as labeled (see the main text for details). "SES" represents the conserved flanking element of "SATR-HERVE-SATR", where SATR represents satellite sequences SATR1 and SATR2. "16p-1", "16p-2", "21q", and "8p" inside the changing core represent the four sequence fragments from the donor loci on chromosomes 16p, 21q, and 8p, respectively. **Bottom**: Using primers indicated above, each type of duplicon was amplified from genomic DNA samples of the 12 primates: A: human or *Homo sapiens *(HSA); B: chimp or *Pan troglodytes *(PTR); C: bonobo or *Pan paniscus *(PPA); D: gorilla or *Gorilla gorilla *(GGO); E: orangutan or *Pongo pygmaeus *(PPY); F: gibbon or *Hylobates leucogenys *(HLE); G: siamang or *Hylobates syndactylus *(HSY); H: rhesus macaque or *Macaca mulatta *(MMU); I: pigtail macaque or *Macaca nemestrina *(MNE); J: tamarin or *Saguinus labiatus *(SLA); K: spider monkey or *Ateles geoffroyi *(AGE); and L: lemur or *Lemur catta *(LCA). The faint band for the 16p-1/21q junction in MMU (lane H of the 3^rd ^gel image on the right) was likely due to nonspecific amplification because the result with another set of primers was negative (repeated multiple times). Additionally, we did not find this junction in the released rhesus macaque genomic sequences [13]. The leftmost and the rightmost lanes are 100 bp and 1 kb ladders respectively (size indicated in Kb).

As described previously [13], four types of duplicons exist in the human genome: Xiao, as well as type I, II and III DAs. Based on their architecture, we designed primers that are specific to each type of duplicon for polymerase chain reaction (PCR) analyses. As shown in Fig. 2a, "standalone Xiaos" (those that are not associated with DAs in the genome) do not have an LTR5B element (solo LTR of HERV-K). This is unlike the "Insertion-ready Xiao" which, based on our previous analyses [13], is likely to have evolved from a "standalone Xiao" by acquiring a HERV-K insertion (which later degenerates to a solo LTR), due to the fact that the donor sequence has neither LTR5B nor HERV-K at the corresponding position. We therefore used the pre-insertion site of LTR5B (HERV-K, to be more precise) to design primers that can only amplify the standalone Xiaos but cannot amplify those Xiao regions inside DAs. Type I DAs appear to have evolved from a LTR5B-containing Xiao via insertion of three sequence fragments derived from chromosomes 16p and 21q into the Xiao core [13] (which is why this Xiao is called "Insertion-ready Xiao" in Fig. 2). Consequently, we designed four pairs of primers flanking both insertion junctions, as well as the two 16p and 21q chimeric sequence fusion junctions (Fig. 2a), to amplify Type I DAs in primate genomes. Compared to type I DAs, type II DAs have an extra HERV-H/HERV-E/HERV-H element (formed by insertion of a HERV-E into an existing HERV-H). Thus, primers were designed flanking the right HERV-E/HERV-H junction, which we found to only exist inside DAs via comparison of the junction sequence with the entire human genome. Finally, given that type III DAs have an additional 100 kb sequence duplication derived from chromosome 8p, primers were designed to span the 16p/8p chimeric sequence fusion junction (Fig. 2a). We also ensured that each primer was not copy-specific and that its sequence was common (with a few base mismatches/gaps allowed) to nearly all copies of a duplicon type.

#### Age of Xiao and three types of DA

With the primers described above, we performed PCR experiments with the primate species indicated in Fig. 1, with each experiment repeated multiple times. As shown in Fig. 2b, Xiao and type I DA were amplified with the genomic DNA of apes but not with that of Old World monkeys, New World monkeys, or the lemur (in most cases, primers designed using the human genomic sequences are able to amplify the orthologous sequence fragments from the ape and Old World monkey genomic DNA, but are unable to achieve the same for the New World monkey and lemur genomic DNA due to larger sequence divergence. Thus, the PCR amplification results with the New World monkey and lemur DNA were expected to be negative for most of our primers. Thus, these species could serve as negative controls in our PCR experiments to a certain extent, while the human served as a positive control). However, type II and type III DAs could be amplified only with the human, chimp, bonobo and gorilla genomic DNA but not with that of other primates. These results indicate that Xiao and type I DA first emerged in the genome between 18 and 25 Myr ago (before the gibbon/siamang diverged from the great apes and after the apes diverged from Old World monkeys [18]), whereas type II and type III DAs emerged between 7 and 14 Myr ago (before the gorilla diverged from the human-chimp common ancestor and after the orangutan diverged from the gorilla-chimp-human common ancestor [18]). This study further refines the previous bioinformatics analyses that compared the released human, chimp, orangutan, rhesus macaque, and marmoset genomic sequences and concluded that Xiao and type I DA emerged within the recent 25 Myr (after the human/ape – Old World monkey divergence [13]) and type II/III DA emerged within the recent 14 Myr (after the human/chimp – orangutan divergence [13]).

The 16p-21q junction primers amplified multiple bands with the human, chimp, bonobo, and gorilla (and possibly orangutan) genomic DNA but only a single band with that of the gibbon and the siamang (Fig. 2b). This implies that polymorphism of this junction is present in the human, chimp, bonobo, and gorilla genomes but absent in the gibbon and siamang genomes. Partial confirmation of this fact was obtained through in-silico PCR analyses (see Materials and Methods) with the published human and chimp genomic sequences, showing amplification of multiple PCR products of approximately 0.6 kb, 0.8 kb and 1.1 kb in size [see Additional file 1]. The only other ape whose genome has been sequenced is the orangutan. Unfortunately, however, the current orangutan release is a draft assembly from 6× whole genome shotgun sequences and contains gaps, which prevent accurate in-silico PCR results from being attained.

By examining the sequences of the in-silico PCR products, we found that the polymorphism is caused by either a 200 bp truncation of the chromosome 16p donor sequence at the fusion junction, or an AluY insertion (300 bp). We also performed in-silico PCR analyses with other primers (Fig. 2a) and found that, overall, the in-silico results agreed with the experimental analyses (Fig. 2b), yielding a single-sized product for Xiao and type II and III DAs but multiple-sized products for type I DAs for the human and chimp genomes [see Table s2 in Additional file 1].

### Age determination of Xiao as well as type I, type II and type III DAs by examining insertion junctions of individual DA/Xiao locus

**Figure 3:**
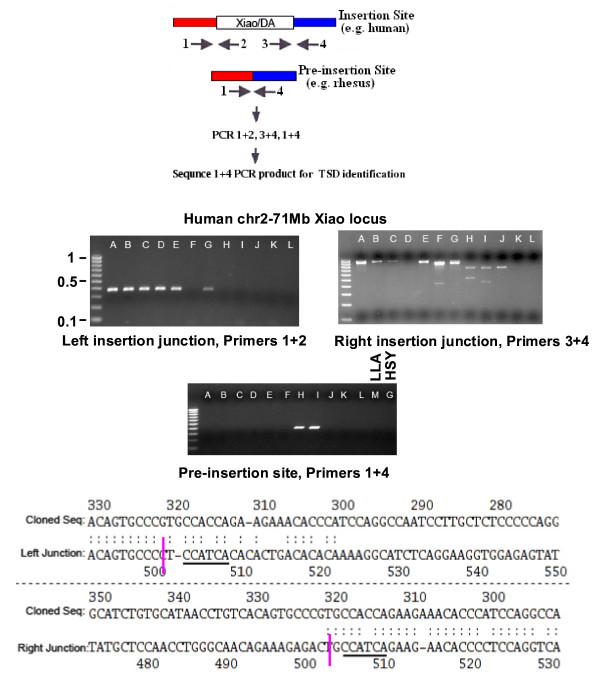
**Copy-specific amplification and TSD determination**. **Top**: For a Xiao or un-rearranged DA locus, three sets of PCR experiments were performed to amplify the insertion (primers 1+2 & 3+4) and pre-insertion (1+4) junctions with the 12 primates. The 1+4 PCR product from humans' closest relatives was sequenced for TSD finding. **Bottom**: The images are the PCR products of the three junctions for chr2-71Mb Xiao. A-L represent primates as specified in Fig. 2, and M represents woolly monkey or *Lagothrix lagotricha *(LLA). The same 100 bp ladders were used as in Fig. 2. The reason that no products were amplified for the left insertion junction for the gibbon and for the right junction for the gorilla (lane F in the left- and D in the right top images) is likely due to variations between the human and the species involved (transposon insertions, sequence mutations, etc.). This is because the other insertion junction and the internal junction (Fig. 2) were amplified, and the pre-insertion junction was not amplified (the bottom image). The small bands in lanes H, I and J of the top right image are likely to be artifacts, because the pre-insertion junction was amplified for H and I. The pre-insertion junction for the siamang (HSY) was the last lane of the bottom image. The preinsertion site PCR product from the pigtail macaque was sequenced ("Cloned Seq") and compared to 500 bp sequences flanking each insertion junction (marked by a vertical pink bar) from the human genome, for TSD identification (CCATCA, underlined).

To determine when each of the 30 DA/Xiao loci identified in the human genome emerged during the course of evolution, we tried to amplify either the two insertion junctions or the pre-insertion junction for a given locus from the genomic DNA of the 12 primate species (Fig. 1) as illustrated in Fig. 3a. We were able to design primers flanking the insertion sites for 25 of the 30 total loci [see Table s1 in Additional file 1]. The remaining 6 loci were found to be located in repeat-rich regions (e.g., near the centromere or the telomere) and, as a consequence, we could not find primer pairs whose sequences were sufficiently unique (i.e., matching many places in the genome).

For loci that are not associated with large inversions (Xiao loci mostly, see reference 13), we performed three sets of PCR experiments to amplify the two insertion junctions and the pre-insertion junction as shown in Fig. 3. In contrast, for loci that are associated with large inversions (mostly DA loci), only the insertion junctions were examined. This is because it is not a straightforward task to pair the primers for amplification of the pre-insertion junction because at least two distinct loci were involved. Also, it is important to note that the inversion might have caused sequence deletions/duplications/insertions, thus complicating the pre-insertion site amplification analysis.

**Figure 4:**
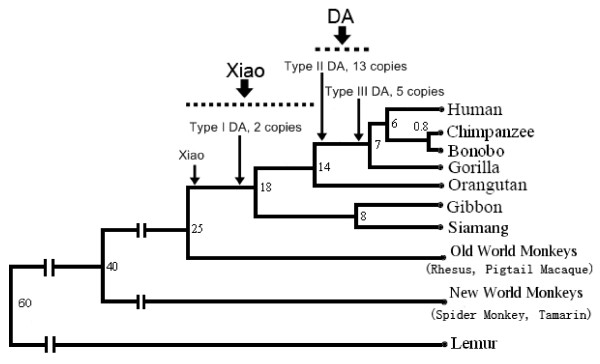
**Age of the DA and Xiao elements**. Thin arrows indicate that Xiao and type I DA first emerged 25-18 Myr ago, whereas Type II/III DA emerged 14-7 Myr ago. Thick arrows indicate that the amplification peak of Xiao and DA occurred during 25-14 and 14-7 Myr ago, respectively. There are a total of 45 copies of Xiao, 2 copies of type I DA, 13 copies of type II DA, and 5 copies of Type III DA. The evolutionary tree of the primates is presented the same way as in Fig. 1.

Each PCR experiment was repeated at least three times in order to confirm the results. In total, at least 2,500 PCR experiments were performed. As summarized in Table 1, the majority of the PCR experiments were conclusive. This required that the results from at least two separate PCR experiments agree with each other for a given PCR reaction, and the results from at least two junctions (out of two insertion junctions and sometimes one pre-insertion junction) agree with each other for a given locus (except 3 loci where we were only able to design primers for one junction, see Table s1 in Additional file 1). This analysis supports conclusions obtained with internal primers as described above. In addition, the results indicate that the Xiao amplification peak was between 25 and 14 Myr ago (after apes diverged from Old World monkeys and before the orangutan diverged from the human-chimp-gorilla common ancestor), whereas the DA amplification peak was between 14 and 7 Myr ago (after the orangutan diverged from the other great apes and before the gorilla diverged from the human-chimp common ancestor) (Fig. 4).

**Table 1:** PCR amplification for each of the DA/Xiao loci found in the human genome^1^

Location (Mb)	Human	Chimp	Bonobo	Gorilla	Orangutan	Gibbon	Siamang	Others ^2^
2p-71, Xiao	Y	Y	Y	Y	Y	Y	Y	N
2q-159, Xiao	Y	Y	Y	Y?^3^	Y	N?	N?	N
3p-8, Xiao	Y	Y	Y	Y	Y	?	?	N
8p-11, Xiao	Y	Y	Y	Y	Y	Y	Y	N
9q-92a, Xiao	Y	Y	Y	?	?	?	?	N
9q-92b, Xiao	Y	Y	Y	Y	?	N?	N?	N
10p-15, Xiao	Y	Y^4^	Y	Y	Y	?	?	N
12q-50, Xiao	Y	Y	Y	Y	Y	Y	Y	N
13q-40, Xiao	Y	Y	Y	Y	Y	Y	Y	N
13q-67, Xiao	Y	Y	Y	Y	?	N	N	N
14q-51, Xiao	Y	Y	N?	Y?	N	N	N	N
21q-32, Xiao	Y	Y	Y	Y	Y	Y	Y	N
3p-75, Type I DA	Y	Y	Y	Y	Y	Y?	Y?	N
3q-127, Type I DA	Y	Y	Y	Y	Y	Y	Y	N
3q/p-131/15^5^, Type II DA	Y	Y	Y	Y	N	N	N	N
4p-4, Type II/I^6 ^DA	Y	Y	Y	Y	N	N	N	N
4p-9, Type II/I^6^/III DA	Y	Y	Y	Y	N	N	N	N
7p/q-6.6/97^5^, Type II DA	Y	Y	Y	Y	N	N	N	N
8p-7-8^7^, Type II/III DAs	Y	Y	Y	Y	N	N	N	N
8p-11-12^7^, Type II/III DAs	Y	N?	?	?	N	N	N	N
11q-67, Type II DA	Y	Y? ^8^	Y?	Y?	N	N	N	N
11q-71, Type III DA	Y	Y	Y	Y	N	N	N	N
12p-8, Type III DA	Y	Y	Y	Y	N	N	N	N

^1 ^Loci not examined (see text) included Xiaos at 2q-95Mb, 3q-113Mb, 11p-17Mb, 13q-45.9Mb and 13q-63Mb, as well as the 11p-3Mb DA. Bioinformatics analyses found all these loci in the chimp genome but not in the rhesus and marmoset genomes. Except for 11p-3Mb DA as well as 13q-45.9Mb and 63 Mb Xiaos, the rest of the loci were found in the current orangutan genome release [13].

^2 ^Others include Rhesus/pigtail/Tamerin/Spider Monkeys and Lemur. In a few cases, non-specific bands (usually not the right size based on the human genome) were amplified with the Old World Monkeys, New World monkeys, and the lemur.

^3 ^"?" indicates that the PCR results were inconclusive (see text), and further analyses are needed to confirm the existence or absence of the locus.

^4 ^This locus was found to be associated with a DA in the published chimp genome.

^5 ^Internal fissions occurred in these DAs and subsequent inversions dispatched broken pieces of DA to several distinct loci [13]. All loci involved were examined by the PCR experiments.

^6 ^We did not find a HERV-H/HERV-E/HERV-H element inside the 4p-4 Mb and -9 Mb DAs in the published human genome, but a previous study [22] found this element at both loci.

^7 ^Several DAs were found in the 8p 7–8 Mb and 11–12 Mb regions.

^8 ^We found a large internal deletion for this DA in the chimp genome (see text).

### TSD identification

Using the strategy indicated in Fig. 3, we sequenced the PCR product of the pre-insertion junction amplified from either the pigtail macaque or the rhesus macaque. We then compared the resulting sequences to sequences flanking each of the two relevant insertion junctions in the human genome, which were determined by aligning the duplicon under study with all other duplicons. For all three Xiao loci that were sequenced, we successfully identified the TSDs [see Table s3 in Additional file 1]. This was consistent with our previous bioinformatics analyses which found TSDs for a majority of Xiao and unrearranged DA loci [13]. These analyses suggest that DA and Xiao may, perhaps, be TEs and that they could have spread throughout the genome via transposition.

### Structural polymorphisms of DA elements between the human and the chimp

At least 29 of the 30 total DA/Xiao loci identified in the human genome were also found in the chimp genome. Proof of this is given by the insertion junction amplification experiments described above (Table 1) and the previous bioinformatics analyses [13]. This means that DA and Xiao have been largely quiet in the human lineage during the last 6 Myr, after the human diverged from the chimp. However, we did find a few large structural variations present between these two apes by comparing the corresponding genomic sequences. For instance, we found that the human 11q-67Mb DA (~280 kb) was degenerated to ~10 kb in the chimp genome, with 270 kb internal sequences deleted [see Fig. s1 in Additional file 1]. On the other hand, the Xiao/DA locus at 15 Mb of chromosome 10 was found to be 252 kb in the chimp genome, but have degenerated to 45 kb in the human genome due to a 200 kb internal deletion of the DA copy [see Fig. s2 in Additional file 1]. Additionally, we could not find the chimp homologues of the human DA/Xiao copies at 11–12 Mb of 8p in the current release of the chimp genome, but we did successfully amplify its external junctions with the chimp DNA sample in our PCR analyses (Table 1) [19]. At this point, we do not know if this is due to large internal deletions of DA/Xiao in the chimp lineage, or to the incompleteness of the current chimp genome release.

## Discussion

Using the knowledge gained by our previous bioinformatics analyses [13] regarding the architecture and genomic locations of the recently discovered composite LTR-retrotransposon-like elements DA and Xiao, we designed experiments to investigate the homologues of the human DA/Xiao elements in primates whose genome has not yet been sequenced. The DA/Xiao pattern results, shown in Fig. 2 and Table 1, seemed to divide the apes into three groups. The first group; including the human, the chimp, the bonobo, and the gorilla; possesses a majority of the Xiao loci as well as DA loci of all three types in their genome. The lesser apes, including the gibbon and the siamang, belong to another group whose genome harbors only a fraction of the Xiao loci and Type I DA loci. The orangutan appears to be between the two groups, having most of the Xiao loci but only Type I DAs. The results indicate that DA and Xiao elements had been most active in great apes but seem to have been quiet in the human lineage during the past 7 Myr (after the human-gorilla divergence). Analysis of the released chimp genome, which did not yield chimp-specific DA/Xiao loci either, supports these results by indicating that DA and Xiao have been most likely quiet in the chimp lineage as well during the past 6 Myr after the human-chimp divergence.

As this analysis only focused on the homologues of the human DA/Xiao loci, we do not know if DA and Xiao have also been quiet in other primate lineages or if they are still active today. To answer this question, it would be helpful to contrast closely related-primates such as the chimp and the bonobo on these elements. Regardless of whether or not these elements are still active today, it will always be informative to search for nonhuman primate-specific DA and Xiao loci, as well as to search for other types of DA and Xiao (i.e., with a different architecture than those shown in Fig. 2) in primates besides the chimp and the human. We did analyze the current orangutan genome release, and did not find orangutan-specific DA/Xiao copies except for one possible Xiao locus. However, this does not necessarily signify that no orangutan-specific DA/Xiao elements are present, because the current release of the orangutan genome is in draft state and some sequences could be missing (especially considering that recent sequence duplications such as DA and Xiao are particularly difficult to be correctly assembled and are usually underrepresented in the draft genome). We will continue this study with newer releases once they become available. Analyzing the gibbon and siamang genomes may provide interesting information, because DA and Xiao emerged immediately before these lesser apes diverged from the great apes. As an increased DA/Xiao activity was observed in the great ape lineages (Fig. 4), it would be useful to find out if amplification of DA/Xiao also peaked in these lesser apes. This would likely yield gibbon/siamang-specific DA/Xiao loci and/or DA/Xiao structures (a possibility indicated by the different amplification patterns between the lesser apes and most great apes with the Xiao-specific primers shown in Fig.2). We hope that these future studies will shed more light on the nature, evolution, and function of these two intriguing elements.

Despite their structural resemblance to composite DNA transposon Tn9 (with a modified HERV-E, which is inactive, replacing the IS element [13]), we have no evidence demonstrating that DA and Xiao are indeed composite LTR-retrotransposons. However, considering the possibility of DA/Xiao being TEs raised by identification of TSDs at a majority of un-rearranged Xiao/DA loci (Table two in reference 13, some shown in Fig. 3 and Table s1), it may not be completely out of the question to hypothesize that DA and Xiao are composite LTR-retrotransposons. If so, we could also speculate that they have relied upon the retrotransposition machinery from active HERV-E elements to move around in the genome in a way that is analogous to a defective virus carrying an oncogene that depends on a helper virus to propagate.

A previous study [20] divided the >50 copies of HERV-E in the human genome into two groups, and reported that group I amplification peaked between 18 and 6–7 Myr ago (after the gibbon-great ape divergence and before the human-chimp divergence). This amplification peak overlapped with those of DA and Xiao (Fig. 4). In addition, similar to the DA and Xiao elements as well, no human-specific or chimp-specific copies of HERV-E were found [21,22], indicating that HERV-E had stopped its activity before the human-chimp divergence. These observations are consistent with the hypothesis described above, stating that DA and Xiao had depended upon the HERV-E machinery to proliferate in the genome. However, the enormous size of DA and Xiao (300 kb and 30 kb respectively) certainly raises questions regarding this hypothesis, as retroviruses including HERVs are below 12 kb and the longest known RNA viruses (corona viruses) are only ~30 kb. Even if the hypothesis is proven to indeed be true, some novel mechanisms which are different from that of HERVs must have been used. This is especially so considering that TSDs identified at the DA/Xiao loci ranged from 4–9 bp with most being 6 bp [13], unlike those of HERVs which are usually 4 bp. Of course, only future studies can clarify these issues.

## Conclusion

To better understand the evolution of the recently discovered composite LTR-retrotransposon-like elements DA and Xiao, we investigated their orthologues in non-human primates whose genomes have not yet been sequenced. These included great apes, lesser apes, Old World monkeys, New World monkeys, and a prosimian. By examining various internal junctions of DA and Xiao in these primate genomes, we found that Xiao and type I DA first emerged between 25 and 18 Myr ago (after the human/apes diverged from the Old Word monkeys and before the gibbon/siamang diverged from the great apes), whereas type II and Type III DAs emerged between 14 and 7 Myr ago (before the gorilla diverged from the human-chimp common ancestor and after the orangutan diverged from the gorilla-chimp-human common ancestor). After investigation of the orthologous sites of the 30 DA/Xiao loci in the human genome, we found that the Xiao amplification peak was between 25 and 14 Myr ago, whereas the DA amplification peak was between 14 and 7 Myr ago. Neither DA nor Xiao seemed to be active in the human and chimp genomes during last 6 Myr. Thus, the activity of DA and Xiao coincided with that of group I HERV-Es during the course of evolution.

The current study led to a more accurate determination of the age of the elements than our previous bioinformatics analyses comparing the released human, chimp, rhesus macaque, orangutan, and marmoset genomic sequences. This information illustrated an evolutionary path with stepwise structural changes for DA and Xiao during the past 25 Myr, and has shed more light on these two intriguing elements.

## Methods

The human (hg18), chimp (panTro2), and rhesus macaque (rheMac2) genomic sequences were obtained from the genome site of the University of California Santa Cruz (UCSC) at . The orangutan contig sequences were obtained from the Washington University Genome Center at genome.wustl.edu. The primers were designed using the Primer3 program at frodo.wi.mit.edu. To ensure that the primer pairs were unique to the duplicons of interest, the primers were compared to the human genomic sequence using the blast program at the Ensemble site at . Usually 500 bp–2 kb amplicons were designed. All the primers used in this study are included in Table s1 in Additional file 1. The non-human primate (Fig. 1) DNA samples were purchased from the Coriell Institute for Medical Research at . The human DNA sample was purified from human embryonic stem cells (BG01).

Each PCR experiment contained 500 nM primers and 5 ng genomic DNA templates that were amplified using the iQ™ Supermix (catalog number 170–8860) from Bio-Rad, which was found to give the most specific and sensitive results after trying various polymerases from several different companies. The PCR reactions were run using an annealing temperature range of 60–66°C and an elongation temperature range of 72–78°C, depending on the primers and the PCR product. In-silico PCR analyses were conducted using the web tools at the UCSC site .

TSD finding: The PCR product of the pre-insertion site of a Xiao locus was cloned into the pGEM vector (Promega) using the AT cloning technology, which was then transformed into the *E. coli *strain Xblue-21 via electroporation. The colonies were screened by PCR analyses and the positive clones were sent to the campus facility for sequencing. The obtained sequences were then compared to the two insertion junction sequences (500 bp flanking the junction that was determined by aligning with other duplicon copies) of the Xiao locus in the human genome using the FASTA sequence alignment program [23] for TSD identification.

## Authors' contributions

XL, JS, and NP conducted the experiments; SZ designed the experiments and wrote the manuscript; JS edited the manuscript. All authors have read and approved the final manuscript.

## Supplementary Material

Additional file 1 **Supporting Materials**. This file provides additional analyses and information to support the conclusions described in the main text. Click here for file
