# Supplementary material for: Stepwise evolution of two giant composite LTR-retrotransposon-like elements DA and Xiao
Source: BMC Evol Biol. 2009 Jun 5;9:128. doi: 10.1186/1471-2148-9-128 (PMC2700803; doi:10.1186/1471-2148-9-128)
Supplement: Additional file 1 — Supporting Materials. This file provides additional analyses and information to support the conclusions described in the main text. [file 1471-2148-9-128-S1.doc]

Table s1: Primer sequences used in the study

| DA/Xiao genomic location (Mb) | Forward | Reverse |
| --- | --- | --- |
| H2-159L* | GAGTTCACGCCAACCTGAGT | AGGTCATCAACCTCGTGGAG |
| H2-159R* | TGGATGAGATTGCAGGTCTG | GTGAACAGGCTACGTGACCA |
|  |  |  |
| H2- 71L | TGCCCACGGATGTAGTGTAA | CAGCATGATTTCGAGAGCAG |
| H2-71R | CCAGCCACCACTTAGCATTT | ACTGAGATCCCGGACATGAG |
|  |  |  |
| H3-8R | GCGACTCTGGCTAATGAGGA | CCAGGATATCTAGCGGGATG |
|  |  |  |
| H3-15R | CCAGCTTTCAAGGACAGGAC | ACCACAACGAAACCTCCATC |
| H3-15L | TGTGCTAGTTGGAGGCCTTT | GACCCAAGCTCGACACTCTC |
|  |  |  |
| H3-131L | CCTGCAGTTGGGATGAATTT | CCCAGATGTCTCCAGATGCT |
| H3-131R | AAAGGTGCGAGTCATCAGGT | ATACCAGGGCATCCTTCCTC |
|  |  |  |
| H3-75L | CTCAATGCTGCGATTCTGAA | GGAGTATCACTGCCCTGGAA |
| H3-75R | GTGTTGAAGTCCGGAACAGC | GAACTCAAGCAGAATGGGAATT |
|  |  |  |
| H3-127R | CCCTAAAGCCCTGAGGAATC | TAATGAGAACCGCACATTGC |
| H3-127L | CGCAGGGTGAATACTCCTGT | GCGAGAAGCGGAAGATGTTA |
|  |  |  |
| H4-4L | CACAAAGAGGCGATGACAAA | TCCTCAACGATGTGATGGAA |
| H4-4R | TGACGCGGATCTCTACACAC | GGGTGTGAGTGGGACTGAGT |
|  |  |  |
| H4-9L | TGGAAGAGAAGAACGGTGCT | AAACACCACGTCGGATCAAT |
| H4-9R | TCAGTCTGCGTGGAACACTC | TCAAGCGTTTGGAGTCCTCT |
|  |  |  |
| H7-97L | TCCCGTAGCATCTTGCCTAT | TGCAAAGAACATCAGGCAAG |
| H7-6L | TAACAGGCCATGTGGAAACC | AGTATCACCGCCCTGGAATAT |
| H7-7R | TGCCATCTTCATCATTGCAT | CCCTGAGCCTGAGACTGAAG |
|  |  |  |
| H8-12DAL | GGGTCAAGCGCTGTTTAAGA | AGCATCTGCTGCAAACTGAA |
| H8-12DA-LastRightJunction | AATTTCATCGTGGGAGAGGA | TACACCCTCCGTGACATGAA |
| H8-11X-LJ | CTGATGACCAGGCACTGCTA | CAAGGTGGCAGTTAGGCATT |
| H8-11X-RJ | CGTAATGGCATCGTCTACCC | ATCTGCTGTTACGCCCATTC |
| H8-7-DR1 | CCGTGTGACGTGGTTCTTAA | TTTGGATTGCCAACAAGTCA |
|  |  |  |
| H9-92aL | CCAGGCTGACCTGAAGAGAT | TTTCCGGTACACGCCATACT |
| H9-92aR | TGGATGAGATTGCAGGTCTG | AAATCACGAACCCAATCTCG |
|  |  |  |
| H9-92bR | ATTGCAACATGGGAGAGGAG | CATGGTGGTTTACCATTGCA |
| H9-92bL | AAAGCCCTTGTCTGCATTTG | TGTACATGCATTGCGACCTT |
|  |  |  |
| H10-15L | CTTAGGATTGCAGCCTCAGG | TGGAAGGACCCAGTTACCAG |
| H10-15R | GAGTCTCGCATTCGTTAGCC | CTTGTGCCTGTTGACATGCT |
|  |  |  |
| H11-71L | TGTGAGGTGGGTCCTAAAGG | GCAAGAGGTGCTGGCTCTTA |
| H11-71R1 | CTGCGACCTTTGCTGTCATA | AGCTGCATTAGCCCTAACGA |
| H11-71R2 | TTCCCCTGCACATTAGGAAG | CCCACTAGGAGGGTTGGAAT |
|  |  |  |
| H11-67L | CTCTAAGCAACACCCATGCA | TGGGACGCTACCTAATGGAC |
| H11-67R | TAAGCCACGAGGAGGTAAGC | ATGGGATAGCACCCACAGAG |
|  |  |  |
| H12-8L | TGGGCAAACATGTGAGTTGT | GGTGGTCTTTCGCTGTTGTT |
| H12-8R | AGGGATTGCGTGAAACAAAC | GACCCTATGGCAGAGAGCTG |
|  |  |  |
| H12-50L | ACATGGTGCTAGGTCCCAAG | ATATTGCAGGCTGTGTGCAG |
| H12-50R | TCAGGAGTATCATCCCGCTAA | ATGTGTGAACACCTCCTGTGA |
| H12-50R2 | TCATGAAAGGCTTGCTGTTG | ACCATTGATCTCCCGAAGTG |
|  |  |  |
| H13-40L | ACAGCAGCCAACCAATCTCT | TTCCTGCAAGGTGGATAGCT |
| H13-40R | AGAGCACCGAATCCTAAGCA | CAGTGTCCACGGAGTAACGA |
|  |  |  |
| H13-67L | CCTCATCTAGCCGCCATTAC | GATACATGGACCGGGACAAC |
| H13-67R | CCTGCGATGCTGGAATTATT | TTCAAGTCTGCATGCCTTTG |
| H13-67R2 | CCACATGCCAGTTAGGTCCT | ACGACTATGGTGGGACCAAG |
|  |  |  |
| H14-51L | TGAAGATCCATATCGCATGG | TTGTTCCCATAACCCTGGAG |
| H14-51R | GAGAGCCACAGGTAGCGAAG | ACATACCTCCTGCGATACGG |
|  |  |  |
| H21-32L | GGCGTCAGTCGCTGTTTATT | GAAATGTATACCCGCGCTGT |
| H21-32R | AGTATCACCGCCCTGGAATA | TTCCGGCGAGATAGACATTT |
|  |  |  |
| **INTERNAL PRIMERS** |  |  |
| H3-126XI (Xiao internal junction) | GCGACTCTGGCTAATGAGGA | CCTGCTATCGTGGGTGAGAC |
|  |  |  |
| H11-67-16-21 junction (Type I DA) | CCCACATGGACACAACTCAG | CACCCATGATAGCAGGGAAT |
| H3-127-16-16 Junction (Type I DA) | TTTGTGGCTTGAGACAGCAC | AGGGAGTGGGTCTCTTAGGG |
| H4-9-16-21 junction (Type I DA) | AGAGAAGGCAGCTGAGGATG | GTCTGCAGTTTGGGATTGGT |
| H11-67-21-21 junction (Type I DA) | CTGAGCCTGGGCATGTTTAT | CCATGCAGGAAAGATGGTTT |
|  |  |  |
| H12-8-HERVH/ER (Type II DA) | TCTGATGGCCACCCTACTTC | TGCCAATATCTCATCCCACA |
|  |  |  |
| H8-12-16-8 junction (Type III DA) | TCCACTGCTGACAGACGTTC | TGGTTAAGGCACCAATGACA |
|  |  |  |

*H represents human; H2 represents human chromosome 2; H2-159L and H2-159R represent the left and right junction of the 159Mb Xiao on chromosome 2 respectively.

**UCSC In-Silico PCR analyses**: as shown below, the in-silico PCR analysis with the 16p-1/21q primers amplified multiple-sized products (578bp, 806bp, 810bp, and 1120bp) from the human genomic sequences. This is consistent with the experimental PCR analysis results shown in Fig. 2. However, for the chimp genome, only a single-sized product of ~810bp was obtained, which is likely due to the sequence divergence between the chimp and human genomes as well as the more stringent PCR condition in the in-silico analyses compared to the actual experiments.

**16p-1/21q primers with the human genome:**

>[chr4:9321391+9322200](http://www.genome.ucsc.edu/cgi-bin/hgTracks?hgsid=103168758&db=hg18&position=chr4:9321391-9322200) 810bp AGAGAAGGCAGCTGAGGATG GTCTGCAGTTTGGGATTGGT

AGAGAAGGCAGCTGAGGATGggaggcctaccgggccctgaacagagagtg

tgtgctgcccagcttcatgaagagccagtgctgcaggtccagaggtgcct

ctttaaagaaagatgccggtttgtcgtagacggtcacagccctgcaatga

aatcatggcggggctattgggggtgctgaagaaaggcctcaggaatagag

gactcagaggctccaggaaaagaaggacgcttggggaatccaagtctcag

acgatgacaagaaaaagccttgcaattacttgggaatgcacagagagatg

tcctaggaccaaaactgcctggaccccctggctggctgggaaggaaactc

tgccccctcctctcccagcttccccaaattttcccataatgttgccaagc

attagtccagcgtgaaggctactttcttctcaaaccactcatttgggtcc

cgcgcccaatgtcacccatcctcagcaaaacaacctttatttccttcttc

ctgctttccagaaagttcccctaaagccctgagaaatcaccgaatgaaag

aggctttttacaaacaggaaacttaagtggagtgccaatacacaacatga

attgcaccaggctgggtctaagataaaaccagactgtggacaacaggaca

gataagaccccccacatggctctgcactgcctgggtctgttattatgtgg

aggaatgtcagtctgttgctcctgtgggtggagctgaagcatgaaccagg

agtcttcatccttagaaagcagttagacagacacctgagaACCAATCCCA

AACTGCAGAC

>[chr8:8136192+8136769](http://www.genome.ucsc.edu/cgi-bin/hgTracks?hgsid=103168758&db=hg18&position=chr8:8136192-8136769) 578bp AGAGAAGGCAGCTGAGGATG GTCTGCAGTTTGGGATTGGT

AGAGAAGGCAGCTGAGGATGggaggcctaccaggccctgaacagagagtg

cgtgctgcccaccttcatgaggagccggtgctgcaggtccagaggtgcct

ctttaaagaaagatgccagcttgtcgtagacggtcacagccctgcaatga

aatcatggcaggactattgcattagtccagcgtggaggctactttctgct

caaaccactcatttgggtcccgtgcccaacgtcacccatcctcagcaaaa

ccaccattatttccttctgcctggtttccagaaagttcccctaaagccct

gaggaatcaccgaatgaaaggggctttttacaaacaggaaacttaagtgg

agtgccaatacacaacatggattgcagcaggctcggtctaagataaaacc

agacggtggacaacaggccaggtaagacccacatggtctgcactccctgg

ttctgttattgtgtggaggaatgtcttagtctgttgctcctgtgggtgta

gctaaagcacaaaccaggagtcctcatccttagaaagcagttagacagac

atctgagaACCAATCCCAAACTGCAGcC

>[chr11:67308341-67309460](http://www.genome.ucsc.edu/cgi-bin/hgTracks?hgsid=103168758&db=hg18&position=chr11:67308341-67309460) 1120bp AGAGAAGGCAGCTGAGGATG GTCTGCAGTTTGGGATTGGT

AGAGAAGGCAGCTGAGGATGggaggcctaccgggccctgaacggagagtg

cgtgccgcccagcttcatgaagagccggtgctgcaggtccagaggtgtct

ctttaaagaaagatgccggcttgtcatagatggtcacagccctgcaatga

aatcatggcaggactattgggagggctgaagaaaggcctcaggaatagag

gactcagaggctccaggaaaagaaggacgcttggggaatccaagtctcag

acgatgacaagaagaagccttgcaatcacttgggaatgcacagagagaca

tcctaggaccaaaactgcctggaccccctggctggctgggaaagaaactc

tgccccctcctctccaggcttccccaaattttcccataatgttgccaagc

attagtccagtgtggaggctactttctgctcaaaccactcatttgggtcc

tgcacccaatgtcacccatcctcagtaaaacaacctttatttccttcttc

ctggtttccagaaagttcccctaaagccctgaggaatcaccgaataaaag

gggctttttacaaacaggaaacttaagtggagtgcctatatgcatcatcg

attgcaccaggctaggtctaagataaaaccagactgtggacaaaaggaca

gataagacccacatggctctgcactgcctgggtctgttattgtgtggagg

aatgtcttagtctgttgctcctgtggatgttgctaaagcacgaaccagga

gttttcatcctttttttttttttttttttttgagacggagtcccactctg

tcgcccaggctggagtgcagtggagcgatctcggctcactgcaagccccg

cctctcgggttcataccattctccttcctcagcctctggagtagctggga

ctacaggcgcccaccaccatgccagctaattttttgtatttttagtagag

acgaggtttcaccgtgttaacaaggatggtctcgatctcctgacctcgtg

atccgcccgtcttggcctcccaaagtgctgggattataagagtaagccac

cgcgcccggcagtcttcatccttagaaagcagttagacagacacctgaga

ACCAATCCCAAACTGCAGcC

>[chr11:3378898-3379703](http://www.genome.ucsc.edu/cgi-bin/hgTracks?hgsid=103168758&db=hg18&position=chr11:3378898-3379703) 806bp AGAGAAGGCAGCTGAGGATG GTCTGCAGTTTGGGATTGGT

AGAGAAGGCAGCTGAGGATGggaggcctaccaggccctgaacggagagtg

cgtgccgcccagcttcatgaagagttggtgctgcaggtccagaggtctct

tgaaagaaagatgctggcttgtcatagacggtcacagccctgcaatgaaa

tcatggcagggctattgggagggctgaagaaaggcctcaggaatagagga

ctcagaggctccaggaaaagaaggatgcttggggaatccaagtctcagac

gatgacaagaagaagccttgcaattacttgggaatgcacagagagatgtt

ctaggaccaaaattgcctggaccccctggctggctgggaaagaaactctg

ccccctcctctcccagcttccccaaattttcccataatgttgccaagcat

tagtccagcgtggaggctactttttgctcaaaccactcatttgggtccca

cgcccaatgtaacccatcctcagcaaaacaacctttatttccttcttcca

cgtttccagaaagttcccctaaagccctaggaatcaccaaatgaaagggg

ctttttacaaacaggaaacttaagtggagtgccaatacacaacatgaatt

gcaccaggctgggtctaagataaaaccagactgtggacagcaagacagat

aagacccacatggctctgcactgcctgggtctgttatggtgtggaggaat

gtcttagtctgttgctcctgtgggtgtagctaaagcacgaaccaggagtc

ttcatccttagaaagcagttagacagacacctgagaACCAATCCCAAACT

GCAGcC

**16-1/21 primers with the chimp genome:**

>[chr4:9439299+9440108](http://www.genome.ucsc.edu/cgi-bin/hgTracks?hgsid=103168758&db=panTro2&position=chr4:9439299-9440108) 810bp AGAGAAGGCAGCTGAGGATG GTCTGCAGTTTGGGATTGGT

AGAGAAGGCAGCTGAGGATGggaggcctaccaggccctgaacagagagtg

tgtgctgcccagcttcatgaagagccagtgctgcaggtccagaggtgcct

ctttaaagaaagatgccggcttgtcgtagatggtcacagccctgcaatga

aatcatggcggggctattgggggtgctgaagaaaggcctcaggaatagag

gactcagaggctccaggaaaagaaggacgcttggggaatccaagtctcag

acgatgacaagaaggagccttgcaattacttgggaatgcacagagagatg

tcctaggaccaaaactgcctggaccccctggctggcttggaaggaaactc

tgccccctcctctcccagcttccccaaattttcccataatgttgccaagc

attagtccagcatgaaggctactttcctctcaaaccactcatttgggtcc

cgcgcccagtgtcacccatcctcagcaaaacaacctttatttccttcttc

ctgctttccagaaagttcccctaaagccctgaggaatcaccgaatgaaag

aggctttttacaaacgggaaacttaagtggagtgccaatacacaacatga

attgcatcaggctgggtctaagataaaaccagactgtggacaacaggaca

gataagaccccccacatggctctgcactgcctgggtctgttattatgtgg

aggaatgtcagtctgttgctcctgtgggtggagctgaagcatgaaccagg

agtcttcatccttagaaagcagttagacagacacctgagaACCAATCCCA

AACTGCAGcC

>[chr11:3404621-3405427](http://www.genome.ucsc.edu/cgi-bin/hgTracks?hgsid=103168758&db=panTro2&position=chr11:3404621-3405427) 807bp AGAGAAGGCAGCTGAGGATG GTCTGCAGTTTGGGATTGGT

AGAGAAGGCAGCTGAGGATGggaggcctaccgggccctgaacggagagtg

cgtgccgcccagcttcatgaagagtcagtgctgcaggtccagaggtgtct

cgaaagaaagatgccggcttgtcatagacggtcacagccctgcaatgaaa

tcatggcagggctattgggagggctgaagaaaggcctcaggaatagagga

ctcagaggctccaggaaaagaaggacgcttggggaatccaagtctcagac

gatgacaagaagaagccttgcaattacttgggaatgcacagagagatgtt

ctaggaccaaaattgccttggaccccctggctggctgggaaagaaactct

gccccctcctctcccagcttccccaaattttcccataatgttgccaagca

ttagtccagcgtggaggctactttttgctcaaaccactcatttgggtccc

acgcccaatgtaacccatcctcagcaaaacaacctttatttccttcttcc

tggtttccagaaagttcccctaaagccctaggaatcaccgaatgaaaggg

gctttttacaaactggaaacttaagtggagtgccaatacacaacatgaat

tgcaccaggctgggtctaagataaaaccagactgtggacaacaggacaga

gaagacccacatggctctgcactgcctgggtctgttattgtgtggaggaa

tgtcttagtctgttgctcctgtgggtgtagctaaagcacgaaccaggagt

ctttatccttagaaagcagttagacagacatctgagaACCAATCCCAAAC

TGCAGcC

**Table s2: In-silico PCR results of internal primers (Table s1) with the human, chimp and orangutan genomes.**

| Duplicon type | Primer | Human(bp)^1^ | Chimp(bp)^1^ | Orangutan(bp)^1^ |
| --- | --- | --- | --- | --- |
| Xiao | H3-126XI | 498 | 494 | *** |
|  |  |  |  |  |
| Type I DA | Left insertion junction H11-67-16-21 | 1177 1187 1189 1190 1191 1192 1193 1196 | 1189 1190 1191 1193 1194 1195 1196 1197 1199 1202 1203 1212 2568 | 1190 1194 1197 |
|  | H3-127-16-16 | 1877 | **** | *** |
|  | H4-9-16-21 | 578 806 810 1120 | 807 810 | 1121 1125 2080 |
|  | Right insertion junction H11-67-21-21 | 1163 1197 | 1156 1213 | *** |
|  |  |  |  |  |
| Type II DA | H12-8-HERVH/ER | 1404 1405 | 1373 1388 | *** |
|  |  |  |  |  |
| Type III DA | H8-12-16-8 | 1011 | *** | *** |
|  |  |  |  |  |
| ***: no PCR product obtained | |  |  |  |

^1^the numbers are the sizes of the amplified products by performing the in-silico PCR analyses with the primers shown in Table s1 on the human, chimp and orangutan genomic sequences, respectively.

| Duplicon | Coordinate | Sequence alignment between cloned  sequence and human duplicon junctions | TSD |
| --- | --- | --- | --- |
| chr14:51M Xiao | hg17 chr14:51280402-51311173 rheMac2 chr7:114792452-114792474 | 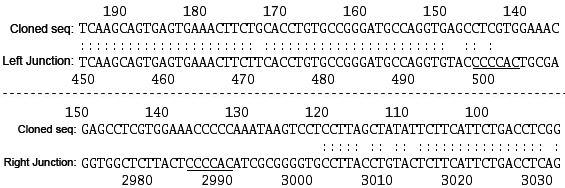 | CCCCAC |
| chr2:71M Xiao | Hg17 chr2:71147690-71196795, Pigtail macaque | 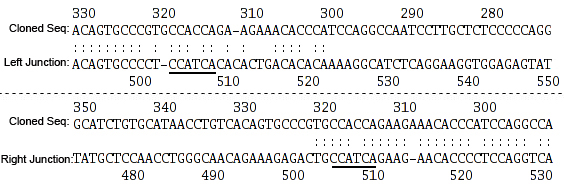 | CCATCA |
| chr13:40M Xiao | Hg17 chr13:40892986-40928830, Rhesus Macaque | 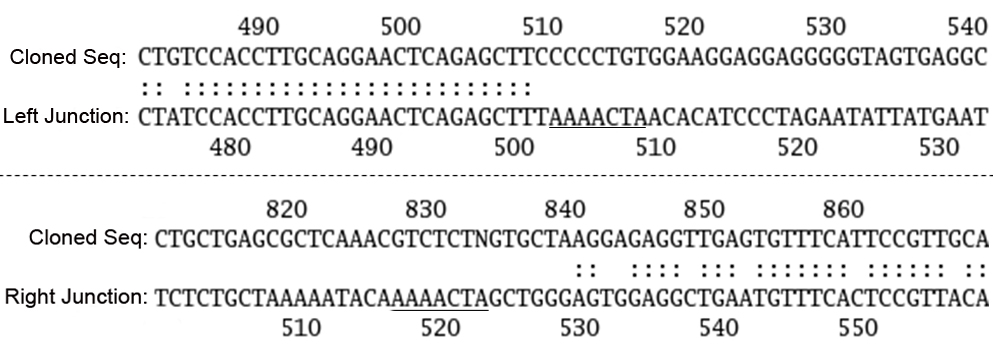 | AAACTA |

**Table s3: TSD Identification***

*The criteria for TSD determination are:

1. The left TSD was near the end of the alignment between the pre-insertion site sequence (PISS) from the non-human primate and the left insertion junction sequence (LIJS) from the human, whereas the right TSD was near the beginning of the alignment between the PISS and the right insertion junction sequence (RIJS). LIJS and RIJS from the human were obtained as described in the main text under “**TSD identification”** and Fig. 3.
2. In the ideal situation, the alignments of the PISS with LIJS and RIJS should overlap over the TSD region and be continuous within the PISS. Thus, the distance between the two alignments within the PISS should be 1bp if taking out the TSD sequence. However, due to changes such as transposon insertions, the distance could be big and we allowed a maximum of 1kb.
3. The right and left TSDs should be exact matches in the human genome; however, sequence mutations were allowed when comparing to the TSD sequence of the non-human primate.
4. TSD should be at least 4bp long.


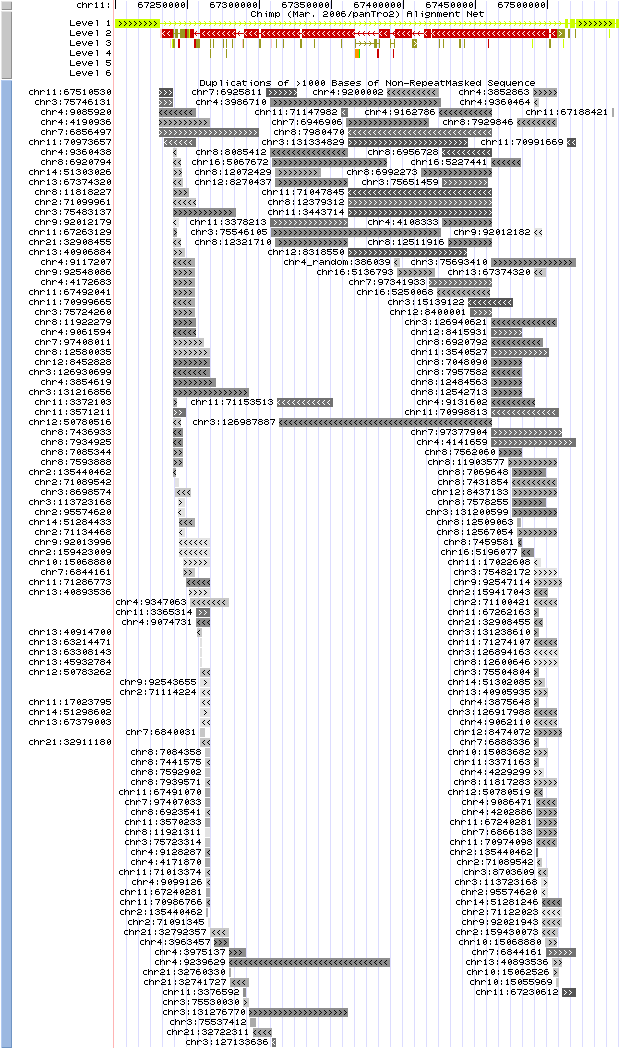


**Fig. s1**: The image of the human chromosome 11-67Mb DA locus (chr11:67,200,190-67,566,710bp) obtained from the UCSC site indicates a ~270kb internal deletion in the chimp homologous region. The top layer shows the sequence coordinate of this human locus in bp. The second layer shows the alignment of this locus with the chimp genomic sequences. The yellowish-green portions at both ends belong to the overall alignment between the human chr11:92549-134448572bp and the chimp chr11:142554-134194740bp. The red bars below indicate that the human chr11:67,252,246-67,507,210bp portion matches to the chimp chr4:9,231,827-9,501,946bp site where a DA copy locates. The third layer shows the alignments of this human DA copy with other DA/Xiao copies found in the human genome.


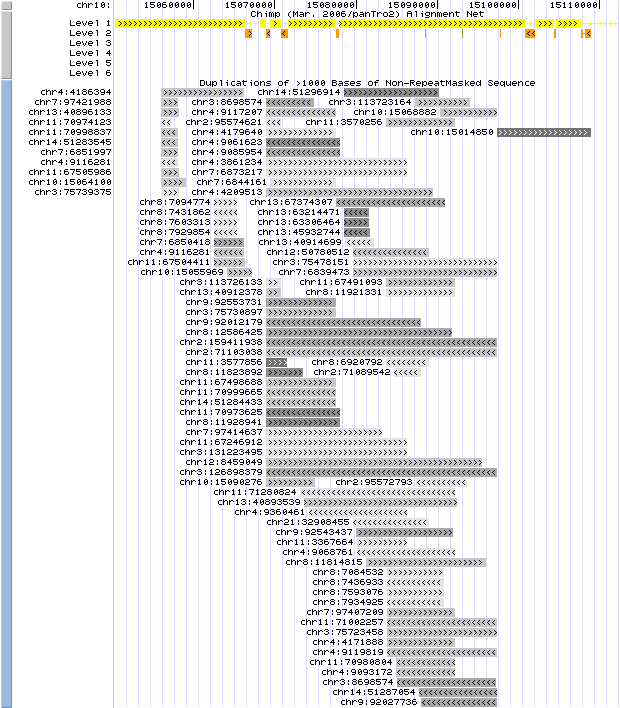


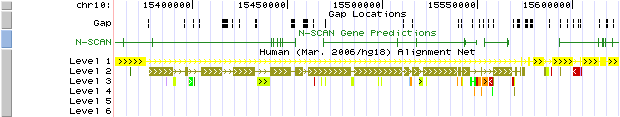


**Fig. s2**: The human chromosome 10-15Mb DA/Xiao locus obtained from the UCSC site is ~45kb (chr10: 15055931-15097361bp). However, the corresponding region in the chimp genome (the following image) is 252 kb (chr10:15,359,507-15,624,298), with the middle portion (chr10:15377512-15572555bp) matching to the human chr3:75478505-75728178bp which belongs to a DA. The images were obtained from the UCSC site and were drawn in the same way as explained in the legend of Fig. s1.
